# Risk prediction of pancreatic cancer using AI analysis of pancreatic subregions in computed tomography images

**DOI:** 10.3389/fonc.2022.1007990

**Published:** 2022-11-09

**Authors:** Sehrish Javed, Touseef Ahmad Qureshi, Srinivas Gaddam, Lixia Wang, Linda Azab, Ashley Max Wachsman, Wansu Chen, Vahid Asadpour, Christie Younghae Jeon, Beichien Wu, Yibin Xie, Stephen Jacob Pandol, Debiao Li

**Affiliations:** ^1^ Biomedical Imaging Research Institute, Cedars-Sinai Medical Center, Los Angeles, CA, United States; ^2^ Gastroenterology, Cedars-Sinai Medical Center, Los Angeles, CA, United States; ^3^ Department of Radiology, Cedars-Sinai Medical Center, Los Angeles, CA, United States; ^4^ Department of Research and Evaluation, Southern California Kaiser Permanente Medical Center, Los Angeles, CA, United States; ^5^ Division of Hematology, Cedars-Sinai Medical Center, Los Angeles, CA, United States; ^6^ Division of Oncology, Cedars-Sinai Medical Center, Los Angeles, CA, United States

**Keywords:** pancreatic ductal adenocarcinoma (PDAC), pancreatic cancer, PDAC prediction, radiomics, pancreatic subregions, abdominal CT scans

## Abstract

Early detection of Pancreatic Ductal Adenocarcinoma (PDAC) is complicated as PDAC remains asymptomatic until cancer advances to late stages when treatment is mostly ineffective. Stratifying the risk of developing PDAC can improve early detection as subsequent screening of high-risk individuals through specialized surveillance systems reduces the chance of misdiagnosis at the initial stage of cancer. Risk stratification is however challenging as PDAC lacks specific predictive biomarkers. Studies reported that the pancreas undergoes local morphological changes in response to underlying biological evolution associated with PDAC development. Accurate identification of these changes can help stratify the risk of PDAC. In this retrospective study, an extensive radiomic analysis of the precancerous pancreatic subregions was performed using abdominal Computed Tomography (CT) scans. The analysis was performed using 324 pancreatic subregions identified in 108 contrast-enhanced abdominal CT scans with equal proportion from healthy control, pre-diagnostic, and diagnostic groups. In a pairwise feature analysis, several textural features were found potentially predictive of PDAC. A machine learning classifier was then trained to perform risk prediction of PDAC by automatically classifying the CT scans into healthy control (low-risk) and pre-diagnostic (high-risk) classes and specifying the subregion(s) likely to develop a tumor. The proposed model was trained on CT scans from multiple phases. Whereas using 42 CT scans from the venous phase, model validation was performed which resulted in ~89.3% classification accuracy on average, with sensitivity and specificity reaching 86% and 93%, respectively, for predicting the development of PDAC (i.e., high-risk). To our knowledge, this is the first model that unveiled microlevel precancerous changes across pancreatic subregions and quantified the risk of developing PDAC. The model demonstrated improved prediction by 3.3% in comparison to the state-of-the-art method that considers the global (whole pancreas) features for PDAC prediction.

## Introduction

Pancreatic Ductal Adenocarcinoma (PDAC) is a lethal cancer that accounts for more than 90% of pancreatic cancer incidences (1–3). At present, PDAC is the 4^th^ key cause of cancer-related deaths (1, 4, 5), with a high expectancy to become the 2^nd^ most by 2030, in both males and females (4, 6, 7). The American Cancer Society anticipates 62, 210 new incidences, and 49, 830 deaths, related to PDAC for the year 2022 in the US (8). The PDAC mostly remains subclinical in the initial stages but progresses rapidly once established. Resultantly, in more than 80% of the cases, cancer has already progressed to later stages by the time of diagnosis (9–12). The negative margin (R0) resection of the PDAC promises long-term survival which is only possible when the cancer is identified at its earliest stages. Treatment, whether surgical or non-surgical, initiated at later stages of the PDAC is associated with poor survival benefits. Although the current overall five-year survival rate of PDAC is barely 11.5%, recent research suggests that detecting PDAC in the earliest stage can increase the survival rate up to 50% (1, 13, 14).

Risk prediction of the PDAC assists in improving the chances of diagnosis at an early stage as follow-up surveillance of high-risk individuals on a regular basis would allow early intervention reducing the chance of missing the initial stages of the disease (15–17). However, since the conventional predictive biomarkers of PDAC lack specificity, risk prediction is challenging. Further, signs and symptoms of pancreatic cancer are either absent or are nonspecific as these are associated with several different diseases (2, 15–18). Factors including the complex location and variability of the pancreas may underlie, in part, the difficulty with an early diagnosis with imaging.

The pancreas undergoes several morphological changes, both locally (e.g., subregional variations) and globally (alterations to the whole pancreas), during the development of PDAC (1, 2). Empirical observations associate PDAC with several preconditioning disorders that usually lead to such morphological and textural changes in the pancreas. For example, complications including IPMN pancreatic tumors (19), distal parenchymal atrophy (20), and pancreatolithiasis (intraductal calculi) (21) gradually increase the heterogeneity of the pancreatic tissue and can potentially be used as a noninvasive risk predictor. Other deformations may include shape and size variations in the pancreas that are consistently associated with ductal dilation (22) and inflammation (23) in the pancreas. However, studies reported that these alterations can be highly subtle and unique to each pancreatic subregion (the term *pancreatic subregion* and *subregion* are used interchangeably). For instance, tumor histology differs across pancreatic subregions (i.e., head, body, and tail) (24, 25) which causes spatial heterogeneity within the pancreas. Also, most of these micro-level variations are difficult to comprehend by visual assessment of abdominal imaging and require computer-based quantification.

AI is the primary choice to perform image-based extensive analysis of such minute alterations and identify potential risk predictors for disease (4, 26, 27). AI systems, as opposed to manual approaches, execute complex tasks without interruption and ensure highly accurate and precise outcomes. In the domain of automated processing and analysis of medical images, AI offers numerous techniques and tools to extract accurate measurements from different structures, identify nonlinear features, and evaluate tissue properties. For prediction modeling, radiomic analysis (28, 29), and machine and deep learning (26, 27, 30) are regarded as the most reliable and common AI approaches.

In our recently published work (31), risk prediction of PDAC was performed using AI analysis of the global features of the pancreas. However, since the morphology of the pancreas was assessed “as a whole”, it remained unknown whether the identified precancerous changes (predictors) were merely the manifestation of local changes that occurred in a specific subregion (presumably where the tumor developed) or all subregions simultaneously adopted such changes.

In this extended study, we thoroughly examined the precursory changes taking place across pancreatic subregions during cancer development and characterized the pancreas that is likely to develop PDAC. A rigorous radiomic analysis of morphological and textural features of three pancreatic subregions (head, body, tail) in the pre-diagnostic abdominal CT scans was performed to identify the features potentially predictive of cancer. Subsequently, a machine learning model was developed that performs risk prediction by automatically classifying the abdominal CT scans into the pre-diagnostic (pancreas at high-risk for cancer) and healthy control (pancreas at low risk for cancer) groups and specifying the subregion of the pancreas that is expected to develop most part of the tumor than its neighboring subregions. To our knowledge, it is the first proposed model to perform the prediction of PDAC based on the subregional analysis of the pancreas. The model remained stable throughout the analysis and outperformed our previous model. The results are promising and encouraging and further validation with a much larger dataset is warranted.

## Materials

### CT imaging for PDAC Diagnosis

Of many imaging modalities, CT plays an important role in the screening for early detection of PDAC. During the initial evaluation of subjects with suspected PDAC, the abdominal CT examination is the common choice to seek primary and secondary signs of cancer. Two institutes, the Cedars-Sinai Medical Center (CSMC) and the Kaiser Permanente Southern California (KPSC) in Los Angeles, collaborated in the proposed study and provided eligible CT scans for analysis. All CT scans were anonymized before transferring to the host institute CSMS. No informed consent was required as the study design is retrospective.

### Datasets for the analysis

**Figure 1:**
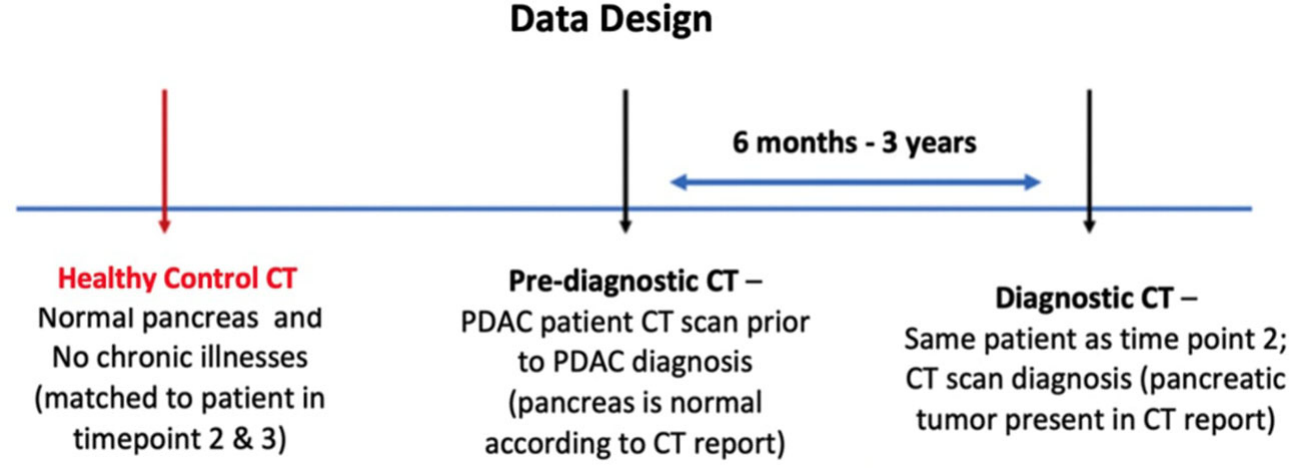
Proposed design of the data for the study. Each case in the dataset consists of three types of abdominal CT scans: Healthy control, Pre-diagnostic, and Diagnostic. The Pre-diagnostic and Diagnostic scans were obtained from the same patient.

The data obtained for the study consisted of contrast-enhanced abdominal CT scans from Diagnostic, Pre diagnostic, and Healthy controls groups. The diagnostic scan belongs to the subject with biopsy confirmed PDAC and observable tumor on the CT scan. These patients do not have any history of pancreatic tumor resection. The pre-diagnostic scan was acquired for the same subject, as in the diagnostic class, 6 months to 3 years before their PDAC was diagnosed. No primary or secondary signs of PDAC were present at the time the pre-diagnostic scan was acquired. The healthy control scan was obtained for a different subject having healthy (‘normal’) pancreas with no history of any pancreatic disorders. The gender and age of each subject in the healthy control class and the year their scan was acquired match those of exactly one unique subject in the pre-diagnostic class to reduce instrumental and morphologic differences, respectively. No subject in the healthy control class developed PDAC within the next 36 months of their scan. The data design of the study is shown in **Figure 1**.

The two institutes obtained 108 CT scans from 72 subjects and were divided into Internal and External datasets. The former consists of 66 scans (22 from each of the three groups) and the latter consists of 42 scans (14 from each of the three groups) from 44 and 28 subjects at CSMC and KPSC respectively. Also, 58 scans (19 diagnostic, 17 pre-diagnostic, 22 healthy control) in the internal dataset and all 42 scans in the external dataset were venous phase images, whereas the rest of 8 scans in the internal dataset belong to multiple phases such as arterial, venous, and connecting phases. The external dataset was used for external validation of the proposed prediction model. **Table 1** provides the split of both internal and external dataset.

**Table 1:** The table provides the split of the total 108 CT scans used in the study.

	Healthy Control scans	Pre-diagnostic scans	Diagnostic scans	Total scans	Number of subjects
**Internal dataset**	22 scans (20 Venous, 2 Arterial)	22 scans (20 Venous, 2 Arterial)	22 scans (18 Venous, 4 Arterial)	66	44
**External dataset**	14 Venous scans	14 Venous scans	14 Venous scans	42	28

### Data reference labeling and preprocessing

For precise measurements of pancreatic features, accurate delineation of the pancreas and the subregions is a prerequisite. The anatomy of the pancreas is complex and requires considerable attention and skills during outlining the pancreas and its subregions. The general shape of the pancreas resembles a hockey stick (J-shaped) structure. On the axial view of an abdominal CT, the pancreas lies across the posterior abdomen. Anatomical subregions of the pancreas consist of the head, body, and tail that appear in the left-to-right order on the axial view of the CT. The head is the expanded medial part lying at the duodenum curve and is attached to the body subregion that connects to a tapered tail subregion. The anteroposterior diameter and the length of the pancreas usually lie between 1 to 3 and 12 to 15 centimeters (32) with the head, body, and tail covering 40%, 33%, and 26% portion of the whole pancreas respectively.

Two experienced radiologists at CSMC manually outlined the boundary of the pancreas and three subregions in all 108 scans using the commercial software ITK-Snap (33). To avoid any prejudgment, findings or information attached to the scans from previous assessments were removed before labeling. A three-step labeling process was performed to ensure labeling consensus. In the first labeling phase, the two readers independently specified the boundary of the whole pancreas and subregions in all scans to limit the inter-reader variability, resulting in 85.4% labeling consistency. In the second phase, both readers were allowed to evaluate each other’s labels and update their original labels which resulted in 97% labeling overlap. Lastly, the 3% labeling conflict in the updated label sets was discussed and resolved with mutual agreement of both graders.

**Figure 2:**
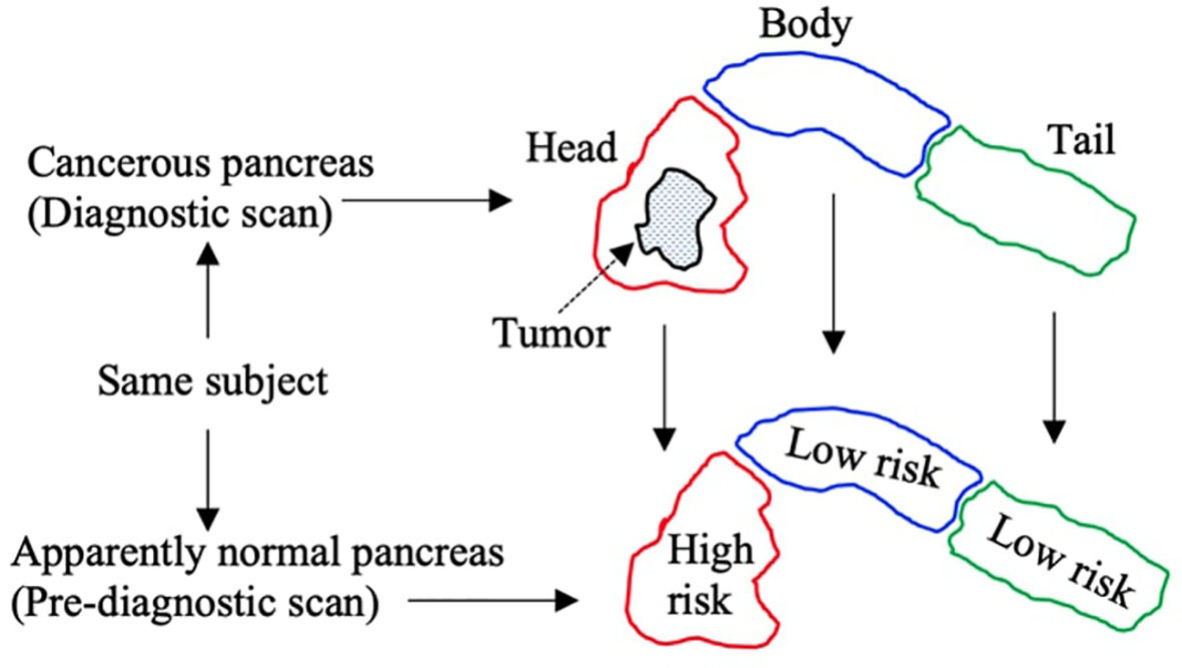
Pictorial description of specifying grades to subregions in pre-diagnostic scans. Tumor is observed in head subregion of diagnostic scans, and so the corresponding head subregion in pre-diagnostic scan is marked ‘high risk’, whereas the rest of subregions are marked ‘low risk’.

In each diagnostic scan, the readers also specified the subregion that contained the greatest amount of pancreatic tumor. This helped grade the subregions in the corresponding pre-diagnostic scans into high-risk and low-risk classes. For instance, if most parts of the tumor were observed in the ‘head’ subregion of the pancreas in a diagnostic scan, then the ‘head’ subregion in the corresponding pre-diagnostic scan was graded as a high-risk subregion, whereas the rest of the neighboring subregions in the same pre-diagnostic scan were graded as low-risk subregions, as given in **Figure 2**. Multiple subregions were graded as high-risk in the same pre-diagnostic scan if the tumor was observed in more than one subregion in the corresponding diagnostic scan. Note that all subregions in the healthy control scans were graded as low-risk subregions. Moreover, from 132 subregions in 44 CT scans (22 healthy control, 22 pre-diagnostic) of the internal dataset, the grading identified a total of 66 and 44 low-risk subregions in healthy control and pre-diagnostic scans respectively, and 22 high-risk subregions in pre-diagnostic scans. For 84 subregions from 28 CT scans (14 healthy control, 14 pre-diagnostic) of the external dataset, the grading identified 42 and 28 low-risk subregions in healthy control and pre-diagnostic scans respectively, and 14 high-risk subregions in pre-diagnostic scans. Furthermore, the pancreas ‘as a whole’ was graded as low-risk and high-risk in healthy control and pre-diagnostic groups respectively.

Each of the 108 scans has 16-bit depth and a slice resolution of 512 by 512 (along the x- and y-axis) and variable z-axis. No preprocessing was performed on any of the scans except the signal intensities in each scan were scaled between 0 and 1.

## Methods

Risk prediction modeling was carried out by thoroughly examining the morphology and the texture of the precancerous subregions to seek predictive features, followed by utilizing these features in a machine learning classifier to automatically characterize the pancreas and subregions into high-risk and low-risk classes for PDAC. The methodology is explained below.

### Radiomic analysis of pancreatic subregions

A large amount of radiomic features were obtained from each of 194 subregions in 66 CT scans (22 healthy control, 22 pre-diagnostic, 22 diagnostic) of the internal dataset, i.e., three sets of features – one for each of the three groups, whereas each set consists of three subsets: one for each of three subregions. Each feature in the set expressed a unique quantifiable property of a subregion that provided information about the spatial relationship of neighboring voxels in predefined proximity (29). To calculate a numerical value for each feature, signal intensities of all 3D pixels specified within a volumetric subregion (all slices) of a scan were considered.

An important aspect of radiomic analysis is to consider the variations in a radiomic feature determined by the three parameters that include the *Kernel* size, the *Angle*, and the *Bin* size (29). Different combinations of these parameters influence the entire analyzation to a high extent. The kernel is the square convolution matrix that specifies the area (proximity) *A* surrounding a voxel *x*, for which the spatial relationships are calculated with its neighbors lying within area *A*. The *Angle* specifies the directions when calculating associations of *x* with its neighbors within the area *A*. The *Bin* size was the number used to discretize the continuous values of voxels in the CT image into their counter parts equal bins to avoid considering two pixels (having too-close signal intensities) any different. Each radiomic feature represented one of the major characteristics of a subregion that includes shape, size, texture, and signal intensity using a unique mathematical expression. Common types of radiomic features considered include first-order statistics (e.g., kurtosis, coefficient of variation, entropy) and higher-order statistics (e.g., contrast, homogeneity, coarseness). With different combinations of three parameters, around 4000 radiomic features from each of 194 subregions were extracted by considering the whole subregion as a single ‘region of interest’.

Using the 132 subregions in 44 CT scans (22 healthy control, 22 pre-diagnostic) in the internal dataset, a pairwise feature comparison between the corresponding subregions (i.e., head-to-head, body-to-body, tail-to-tail) was performed to identify the features that were significantly different between high-risk and low-risk subregions. For example, the extracted features from all low-risk head subregions in the internal dataset were compared with the same set of features extracted from all high-risk head subregions in the internal dataset. About 3.5% of the extracted features showed significance (found potentially predictive) at a p-value of 0.05 in the statistical t-tests—supporting the core hypothesis about the presence of precancerous changes occurring locally within the subregions undergoing tumor development. Note that the only purpose of considering the features extracted from the 66 subregions in 22 CT diagnostic scans in the internal dataset during the analysis was to help sub-selecting the predictive features that are highly stable and do not become insignificant when pre-diagnostic and diagnostic scans are mixed.

### Risk prediction of PDAC

The significant features (predictors) identified through the subregional analysis were used to perform automated risk prediction of PDAC by classifying the pancreas into either low-risk or high-risk categories. The criteria set to perform binary classification was to mark the pancreas as low-risk if none of its subregions was classified as high-risk, whereas the pancreas was marked as high-risk, if at least one of its subregions was classified as high-risk. A misclassification is counted if a) the classifier marks one or more subregions as high-risk in a healthy control scan, or b) the classifier identifies a high-risk subregion as low-risk in the pre-diagnostic scan or vice versa.

**Figure 3:**
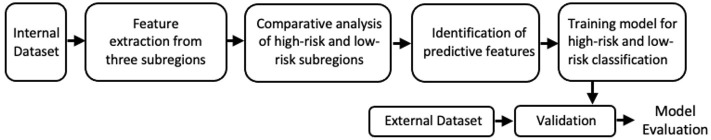
The major steps performed in the analysis and prediction process.

The Naïve Bayes (NB) model was trained for binary classification in conjunction with the Recursive Feature Elimination (RFE) (34, 35) method in which the RFE method eliminated the weak features using different combinations of identified predictors while maximizing the overall training accuracy based on the given classification criteria. Of note, the RFE was prespecified to select up to the seven best features to avoid overfitting the NB classifier. The NB-RFE identified seven features (Long-run low grey-level emphasis, Gaussian left polar, Inverse gaussian left polar, Inverse cluster shade, Inverse cluster prominence, Inverse cluster tendency, Short-run low grey-level emphasis) as the best predictors for the classifier to get the maximum classification accuracy during training the model on all the 44 CT scans (132 subregions) of the internal dataset. The external validation of the trained model was then performed using 24 CT scans (84 subregions) of the external dataset. An overview of the prediction process is provided in **Figure 3**.

## Results

Model performance was evaluated in terms of classification accuracy, sensitivity, and specificity. The classification accuracy was calculated as the total number of correctly classified scans (both healthy control and pre-diagnostic) to the total number of scans input to the NB classifier. The sensitivity is the true positive rate which refers to the total number of correctly classified pre-diagnostic scans (high-risk pancreas) to the total number of pre-diagnostic scans input to the NB classifier. Whereas the specificity is the true negative rate which refers to the total number of correctly classified healthy control scans (low-risk pancreas) to the total number of healthy control scans input to the NB classifier.

The mean classification accuracy achieved on the training data (internal dataset) was 93% (41/44), i.e., the number of correctly classified scans to the total number of scans observed. The external validation of the classifier was performed using the 56 subregions in 28 scans (14 healthy control and 14 pre-diagnostic) in the external dataset. The validation achieved the mean classification accuracy of 89.3% (25/28), with the sensitivity and specificity reaching 86% and 93% respectively, as given in the confusion matrix **Table 2**.

**Table 2:** Confusion matrix for classification of 28 CT scans of the external set consisting of 14 from each of Healthy control and Pre-diagnostic group.

	True Healthy	True Pre-diagnostic
Predicted Healthy	13	2
Predicted Pre-diagnostic	1	12

Numbers in the orange blocks show true positives.

Compared to the performance of our previous prediction system (31) which produced 86% classification accuracy, the proposed model demonstrated improved accuracy by 3.3%. Also, it was empirically observed that the inter-variability between the features extracted from corresponding ‘low-risk’ subregions identified in healthy control and pre-diagnostic scans was significantly low at a p-value of 0.05. This supports our primary hypothesis that the precancerous changes predominately occur locally and are specific to the subregion within which the tumor is likely developing. Also, the 95% confidence interval (CI) achieved in the current study is 78-100, showing modest improvement on the lower bound of the CI obtained in the previous study (i.e., 73-99). Further improvement in the current CI was possible if the model training was not enforced to use a fixed limited number of predictors to avoid model overfitting.

Moreover, the radiomic analysis infers that it is essentially the texture of the pancreas that changes locally and appears abnormal on a CT scan during cancer development. These textural changes are the possible indication of the stage the underlying healthy cells are transitioning into tumor cells (e.g., the tumorous region turns more hypointense than the non-tumorous peripheral region on a CT image). Furthermore, the shape of the whole pancreas (in healthy and pre-diagnostic scans) and subregions (belong to high-risk and low-risk classes) was observed indifferent, partly because the shape of the pancreas is highly irregular in general. However, the size of the high-risk subregions was observed slightly higher than their corresponding low-risk subregions, though not significantly different to be considered a stable predictor.

## Discussion

### Clinical significance of PDAC prediction using CT imaging

The Centers for Disease Control and Prevention reports that 7 million patients with abdominal pain visit to ER in the US each year. These patients undergo CT examinations as per the standard care protocol. The initial evaluation of these scans assists clinicians to identify the underlying cause of abdominal pain. Though the scans of majority of these patients do not present any signs of cancer at this stage, some ultimately develop PDAC in coming years. These pre-diagnostic scans, even with no prominent signs of cancer, are clinically useful as these might contain significant morphological signatures of early biological adaptations associated with cancer. AI techniques can efficiently assist in identifying these signs and forecasting cancer incidence for the future. However, AI-based exploration of precancerous signs is challenged by data scarcity as the PDAC has a low prevalence. In this retrospective study, we examined the quantitative difference of the CT-based features between pre-diagnostic and healthy control scans. The study allowed quantitative analysis of the subregional changes that occurred in the precancerous or pre-symptomatic pancreas and helped reduce limitations of low prevalence and low cancer yield in prospective studies as half of the subjects have cancer.

The unique data structure designed for this study is the foundation of the proposed prediction model as it allowed examining precancerous changes retrospectively. Although the overall prevalence of PDAC is significantly low, the percentage of enrolled subjects who were at the preclinical stage was set to 50% to reduce the risk of class imbalance during model development. Also, most of the literature considers that the duration of 6-36 months between the pre-diagnostic and diagnostic scan is a reasonable window to seek early signs.

Also, most of the scans used for mode training and testing are portal venous phase. It is because tumors slowly uptake contrast whereas the venous phase provides the optimal view of the tumor edges and is thus considered the most valuable phase for PDAC diagnosis. Also, viewing of the vasculature passing across or alongside the pancreas is optimized in this phase. Changes occurring to the vasculature during PDAC development can be quantified and used as potential predictors. Nevertheless, other phases also provide valuable information during PDAC screening and treatment. For example, the arterial phase provides a unique value when seeking lesions or during surgical treatment of PDAC when the arteries are encased or distorted by the pancreatic tumor. Thus, including multiphase scans in the model training helped identify highly stable predictors to ensure the model is sufficiently robust.

In accordance with the evidence provided, the proposed research work assures the appropriate blend of imaging type, feature analysis, and modeling techniques to address the challenges of prediction and elevate the chances of cancer diagnosis in the earliest stage. To our knowledge, it is the first automated system developed that predicts the PDAC by identifying early signs through analyzing the precancerous irregularities occurring within pancreatic subregions using CT scans. The proposed model not only demonstrated improved prediction accuracy to existing models but also enabled the system to identify subregions that are at higher risk of developing tumors.

### Significance of the subregional analysis

Several studies suggest that tumor development differs across pancreatic subregions (Head:*H* , Body: B, Tail:*T*) in terms of histology, presentation, and symptoms (24, 25, 36–39). For instance, tumors in the head are mostly non-squamous, whereas the body and tail tumors are usually squamous. This results in spatial heterogeneity and various discrepancies across the pancreatic sub-regions; such as tumor presentation (e.g., head tumors are usually well-differentiated and less aggressive than those in body/tail), related symptoms (head tumors: unexplained weight loss, body tumors: pain in the upper abdomen, tail tumors: pain in the lower abdomen), sensitivity to drugs (head tumors are highly responsive to Gemcitabine regimen and less responsive to Fluorouracil regimen, whereas the body and tail tumors are vice-versa), and the different rates of incidence (*H*: 71%, *B*: 13%, *T*: 16%), metastasis (*H*: 42%,*B*: 68%, *T*: 84%), %), 2-year survival (*H*: 44%, *B*: 27%, *T*: 27%), and resection (*H*: 17%,*B*: 4%, *T*: 7%) (24, 25, 36–39).

This study examined the subregional changes in the precancerous pancreas and enabled automated identification of subregions undergoing tumor development. Knowledge of the location of likely tumor will not only alert clinicians/radiologists to pay attention to certain regions of the pancreas to avoid misdetection of PDAC at an early stage but also enhance the overall management of PDAC by helping determine more appropriate and effective treatment, improving forecasting of the treatment outcome, planning better resection, and ultimately increasing the overall survival rate.

### Improvement to previous model

In our previous study (31), we proposed the first model for risk prediction of PDAC using AI analysis of the morphology of the ‘whole pancreas’. The current study has three major new contributions which were not included in the previous study (31): a) investigation performed to identify whether all three subregions concurrently adopt precancerous changes, or the changes are predominant (or only occur) in the subregion where the tumor is likely developing, b) analysis performed to identify new CT-based predictors and improved the prediction (in terms of model accuracy), and c) trained the model to specify the subregion that is at highest risk of developing most part of the tumor or where the tumor will likely originate.

### Study limitations and future work

Due to the low prevalence of PDAC, the eligible pre-diagnostic CT scans were found rare in the data archives of the CSMC and KPSC. Limited training data may have increased the chance of overfitting during model development. Another limitation is the insufficiency of pre-diagnostic CT scans of non-venous CT phases. Also, since the incidence of PDAC in the general population is fairly low, the model specificity of 93% still requires further enhancement to avoid too many false-positive cases. The aim of the study was to present the proof of the concept which encourages the collection of larger datasets including the information on non-imaging factors associated with risk of PDAC from the repositories of different institutes for substantial training and validation of the proposed prediction model. With sufficient data, the biological interpretation of predictive image features and their correlation with genetics would be achievable. A model trained on large data will improve model specificity and will efficiently assist in future prospective research on detecting PDAC at the initial stages.

## Conclusion

The current study presented the findings of the AI analysis of precancerous changes that occurred across three subregions of the pancreas using pre-diagnostic abdominal CT scans. The study concluded that the pancreas adopts textural changes during PDAC development, predominantly within the subregion undergoing tumor development, potentially regarded as a ‘high-risk’ subregion. A first model was built that performed risk quantification of PDAC using the identified textural changes as potential predictors and characterized the pancreas into ‘high risk’ and ‘low risk’ for PDAC classes. The model also specified the subregion that is likely to develop the tumor, which can potentially assist in improving early diagnosis, treatment planning, forecasting treatment outcome, and overall disease management. The proposed model demonstrates a 3.3% improved prediction when compared with the existing prediction model that considers the global changes occurring in the whole pancreas during PDAC development. The results of this preliminary study are promising and encouraging to further validate the model on a large dataset.

## Data availability statement

The original contributions presented in the study are included in the article/supplementary material. Further inquiries can be directed to the corresponding authors.

## Author contributions

DL, SP, SG, BW, CJ, and TQ were responsible for the conception and design. LW, LA, AW, WC, and VA performed data extraction and data labeling. SJ and TQ conducted basic investigation, performed statistical analysis, developed prediction model, and wrote original manuscript. DL performed quality control of this study. All authors contributed to the article and approved the submitted version.

## Funding

NIH R01 CA260955.

## Conflict of interest

The authors declare that the research was conducted in the absence of any commercial or financial relationships that could be construed as a potential conflict of interest.

## Publisher’s note

All claims expressed in this article are solely those of the authors and do not necessarily represent those of their affiliated organizations, or those of the publisher, the editors and the reviewers. Any product that may be evaluated in this article, or claim that may be made by its manufacturer, is not guaranteed or endorsed by the publisher.
